# A real-world study of antifibrotic drugs-related adverse events based on the United States food and drug administration adverse event reporting system and VigiAccess databases

**DOI:** 10.3389/fphar.2024.1310286

**Published:** 2024-02-23

**Authors:** Menglin He, Taoran Yang, Jian Zhou, Rurong Wang, Xuehan Li

**Affiliations:** Department of Anesthesiology, West China Hospital, Sichuan University, Chengdu, Sichuan, China

**Keywords:** adverse events, adverse drug reaction, pharmacovigilance, pirfenidone, nintedanib

## Abstract

**Objectives:** This study aims to investigate adverse events (AEs) and adverse drug reactions (ADRs) associated with pirfenidone and nintedanib, two antifibrotic drugs used to treat idiopathic pulmonary fibrosis (IPF).

**Methods:** Reporting odds ratio (ROR) and proportional reporting ratio (PRR) analyses were conducted to assess the association between these drugs and signals at both the preferred term (PT) and system organ class (SOC) levels.

**Results:** 55,949 reports for pirfenidone and 35,884 reports for nintedanib were obtained from the FAERS database. The VigiAccess database provided 37,187 reports for pirfenidone and 23,134 reports for nintedanib. Male patients and individuals over the age of 65 were more likely to report AEs. Gastrointestinal disorders emerged as the most significant signal at SOC level for both drugs. Furthermore, nausea, diarrhoea, and decreased appetite were observed at the PT level. We further identified notable signals, including hemiplegic migraine for pirfenidone and asthenia, constipation, and flatulence for nintedanib, which were previously unknown or underestimated ADRs.

**Conclusion:** This study has identified AEs and ADRs associated with pirfenidone and nintedanib, confirming that the majority of the corresponding label information indicates relative safety. However, it is essential to take unexpected risk signals seriously, necessitating further research to manage the safety profiles of these drugs.

## 1 Introduction

Idiopathic pulmonary fibrosis (IPF) is a chronic and progressive interstitial lung disease (ILD) characterized by the excessive development of fibrotic tissue in the lungs, leading to impaired respiratory function, an increased risk of respiratory failure, and complications causing mortality (Lederer and Martinez, 2018). Globally, IPF affects approximately 3 million individuals, with a median survival ranging from 2 to 3 years (Quinn et al., 2019). Despite extensive research efforts, the exact etiology of IPF remains elusive (Podolanczuk et al., 2023). Nonpharmacologic interventions are pivotal in improving the overall wellbeing and quality of life for IPF patients, enabling them to lead healthier lives (Liu et al., 2022). Moreover, among the limited treatment options currently available, nintedanib and pirfenidone, two antifibrotic drugs, are recommended as first-line treatment for IPF due to their promise in slowing progression, preserving lung function, and improving patient outcomes (Flaherty et al., 2018).

Pirfenidone exerts regulatory effects on fibrogenic growth factors, particularly transforming growth factor (TGF)-β1. It mitigates fibroblasts proliferation and differentiation, as well as the synthesis of collagen, fibronectin, and other extracellular matrix (ECM) components (Ruwanpura et al., 2020). Clinical evidence shows that pirfenidone can reduce the decline in forced vital capacity (FVC) and improve progression-free survival in IPF patients (Takehara et al., 2023). Firstly approved for IPF treatment in Japan, pirfenidone has gained clinical recognition in America and Europe. Nintedanib, a triple angiokinase inhibitor, is another disease-modifying therapy approved for the treatment of IPF. It targets key fibrogenesis pathways, including platelet-derived growth factor (PDGF), fibroblast growth factor (FGF), and vascular endothelial growth factor (VEGF) signaling (Flaherty et al., 2019). Clinical trials, such as INPULSIS trials, have demonstrated that nintedanib reduces the annual decline in FVC and slows IPF progression, leading to its first approval as a treatment option for IPF in the United States in 2014 (Crestani et al., 2019).

However, despite their therapeutic benefits, drugs can pose unforeseen harm in the form of adverse events (AEs), affecting effects of drug, prognosis and patients outcome (Feagins et al., 2023). In addition, adverse drug reactions (ADRs), defined as the unexpected and harmful response that occur during drug administration, should also be taken seriously (Basile et al., 2023). Pirfenidone administration has been linked to gastrointestinal symptoms, skin rashes and significant liver function abnormalities, necessitating regular monitoring (Lancaster et al., 2017). Moreover, the occurrence of ADRs related to pirfenidone demonstrates a dose-dependent relationship and can be ameliorated through adjustments in mode and dose of administration (Kang et al., 2023). The reported common ADRs associated with nintedanib were diarrhea, bronchitis, nasopharyngitis, and cough (Li et al., 2023). Tthe occurrence of these ADRs in hospitalized patients not only imposes financial burden but also prolongs their hospital stay, and in severe cases, poses life-threatening risks. Therefore, it is crucial for clinicians to have a thorough understanding of these potential ADRs, as close monitoring and prompt management can help mitigate the impact of these ADRs.

While clinical trials play a crucial role in establishing the efficacy of novel medications and identifying common ADRs, they may not capture all real-world scenarios due to the potential for rare and severe events that may only emerge after widespread administration of the drug in clinical settings. Fortunately, these limitations could be solved with the emergence of pharmacovigilance (PV) analysis, which involves monitoring and evaluating the safety profile of pharmaceutical products. Data sources such as the FDA Adverse Event Reporting System (FAERS) and the World Health Organization’s VigiAccess database enable the collection, analysis, and assessment of ADRs and other medication-related safety issues on a population level (Rong et al., 2023).

In the present study, we conducted a statistical analysis of the data obtained from the FAERS and VigiAccess databases to identify the AEs and ADRs signals associated with pirfenidone and nintedanib. In addition, we categorized AEs for both drugs and compared the risk of ADRs between them. These findings provide valuable insights into the safety of clinical medication and support evidence-based decision-making in drug selection.

## 2 Methods

### 2.1 Data source

OpenVigil 2.1 (https://openvigil.sourceforge.net/), a widely-used online tool in pharmacovigilance research, was utilized for data mining and pharmacovigilance data analysis (Böhm et al., 2023). In this study, we employed this online tool to retrieve data from the FAERS database, covering the period from 1 January 2015, to 1 January 2023 for pirfenidone and nintedanib, in accordance with their approval dates. Various pharmaproduct names of pirfenidone (such as “esbriet”, “pirfenidone aet”, “pirfenidone axunio”, “pirfenidone viatris”, “truemed group llc”) and “ofev” or “vargatef” for nintedanib were used as search terms. Only primary suspect roles were considered for the analysis. Furthermore, VigiAccess database, a valuable resource for healthcare professionals and researchers, was also utilized to search for data related to these two drugs.

### 2.2 Data standardization

AEs documented in the FAERS database underwent systematic categorization and encoding using the Medical Dictionary for Regulatory Activities (MedDRA) terminology. Each individual record was assigned a preferred term (PT), which can be further categorized into various systems based on the System Organ Class (SOC).

### 2.3 Statistical analysis

Disproportionality analysis, a data mining algorithm known for its high sensitivity and effectiveness in mitigating various biases, was extensively employed in the global monitoring of ADRs. Each signal identified through this approach represents a statistical association between a specific drug and an AE. For our study, a combination of proportional reporting ratio (PRR) (Evans et al., 2023) and reporting odds ratio (ROR) (Rothman et al., 2023), both known for their heightened sensitivity, were utilized in our signal mining process. The screening criteria for PRR included: N ≥ 3; χ2≥4; PRR≥2. As for ROR, N ≥ 3 and a lower limit of 95% CI > 1 was set as criteria. AEs that met both PRR and ROR criteria underwent further analysis.

## 3 Results

### 3.1 Characteristic analysis of AE reports

**FIGURE 1:**
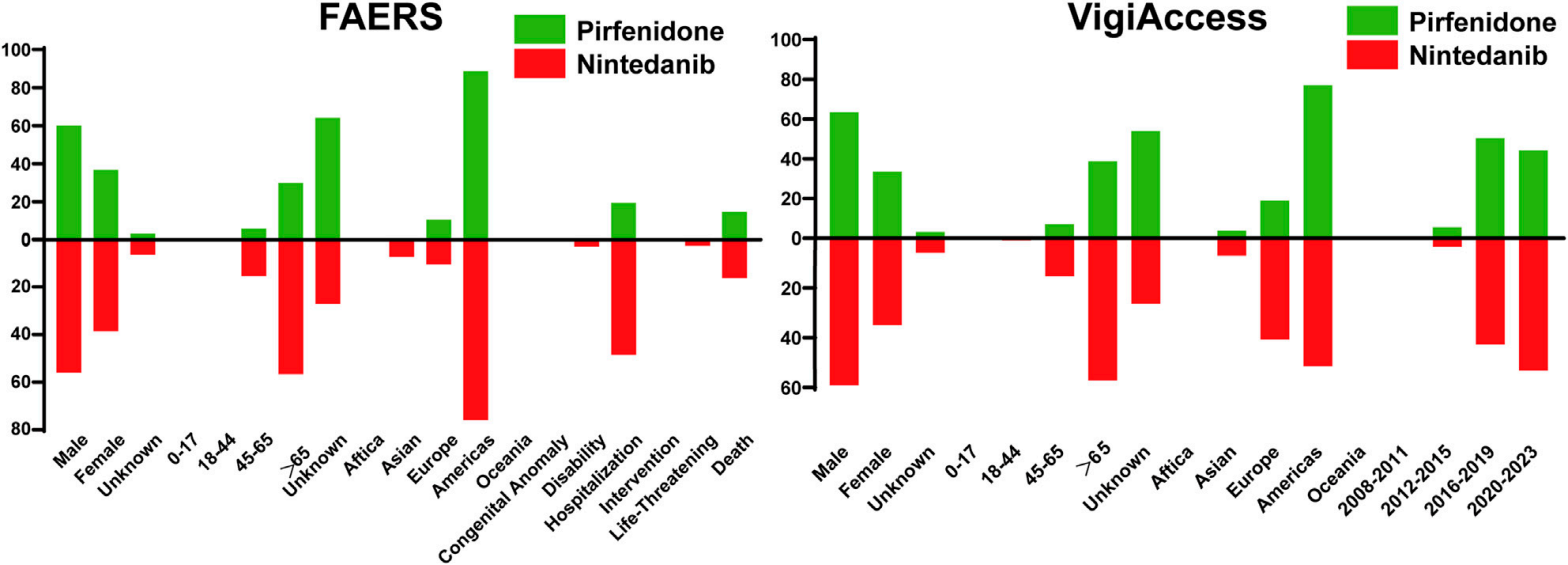
Characteristic analysis of AE reports in the FAERS and VigiAccess databases.

The characteristic results were depicted in Figure 1. The FAERS database contained a total of 55,949 AE reports associated with pirfenidone and 35,884 reports associated with nintedanib, surpassing the corresponding figures reported in the VigiAccess database (37,187 and 23,134). Furthermore, male patients predominated over females for both drugs in both databases (Pirfenidone: 60.09% vs 36.73% in the FAERS database; 63.36% vs 33.45% in the VigiAccess database, Nintedanib: 55.98% vs 38.66% in the FAERS database; 59.17% vs 34.94% in the VigiAccess database). Concerning age distribution in the FAERS database, patients over the age of 65 were more likely to reporting AE, constituting 29.88% (16,716) for pirfenidone and 56.65% (20,330) for nintedanib. Similar results were shown in the VigiAccess database (14,409, 38.75%; 13,245, 57.25%). However, more than half of cases (20,047, 53.91%) lacked age information for pirfenidone. The main reporting continent was the Americas for both drugs in both databases. Among all cases, the most severe outcome was hospitalization (10,869, 19.43% for pirfenidone; 17,433, 48.58% for nintedanib). Additionally, AE reports for these drugs showed an increasing trend over the years.

### 3.2 Signal detection at PT level

**FIGURE 2:**
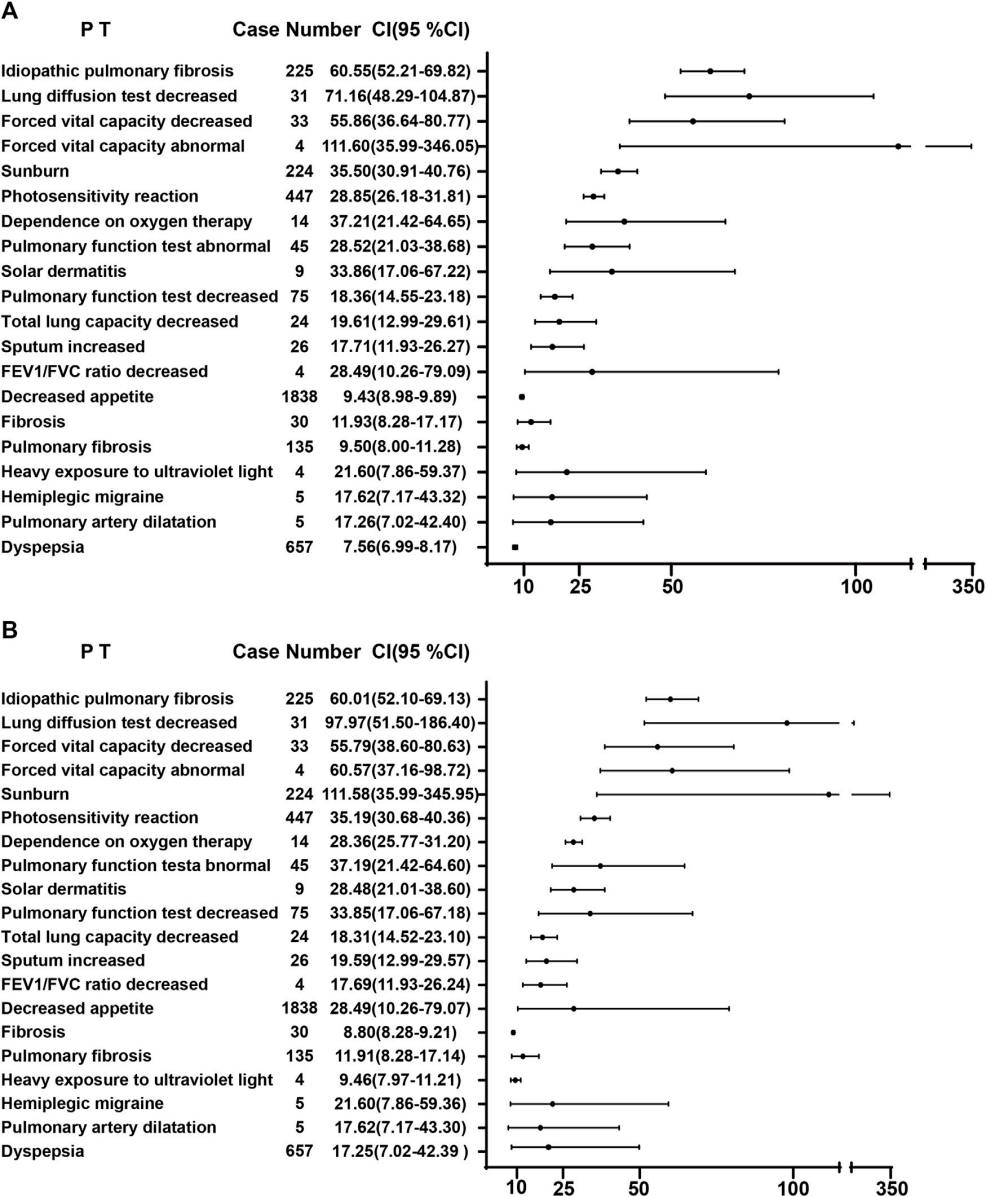
The ROR **(A)** and PRR **(B)** disproportionality analysis of signals at PTs level associated with pirfenidone in the FAERS database. ROR, reporting odds ratio; PRR, proportional reporting ratio; PTs, preferred terms; FAERS, Food and Drug Administration Adverse Event Reporting System; 95% CI, 95% credibility interval.

**FIGURE 3:**
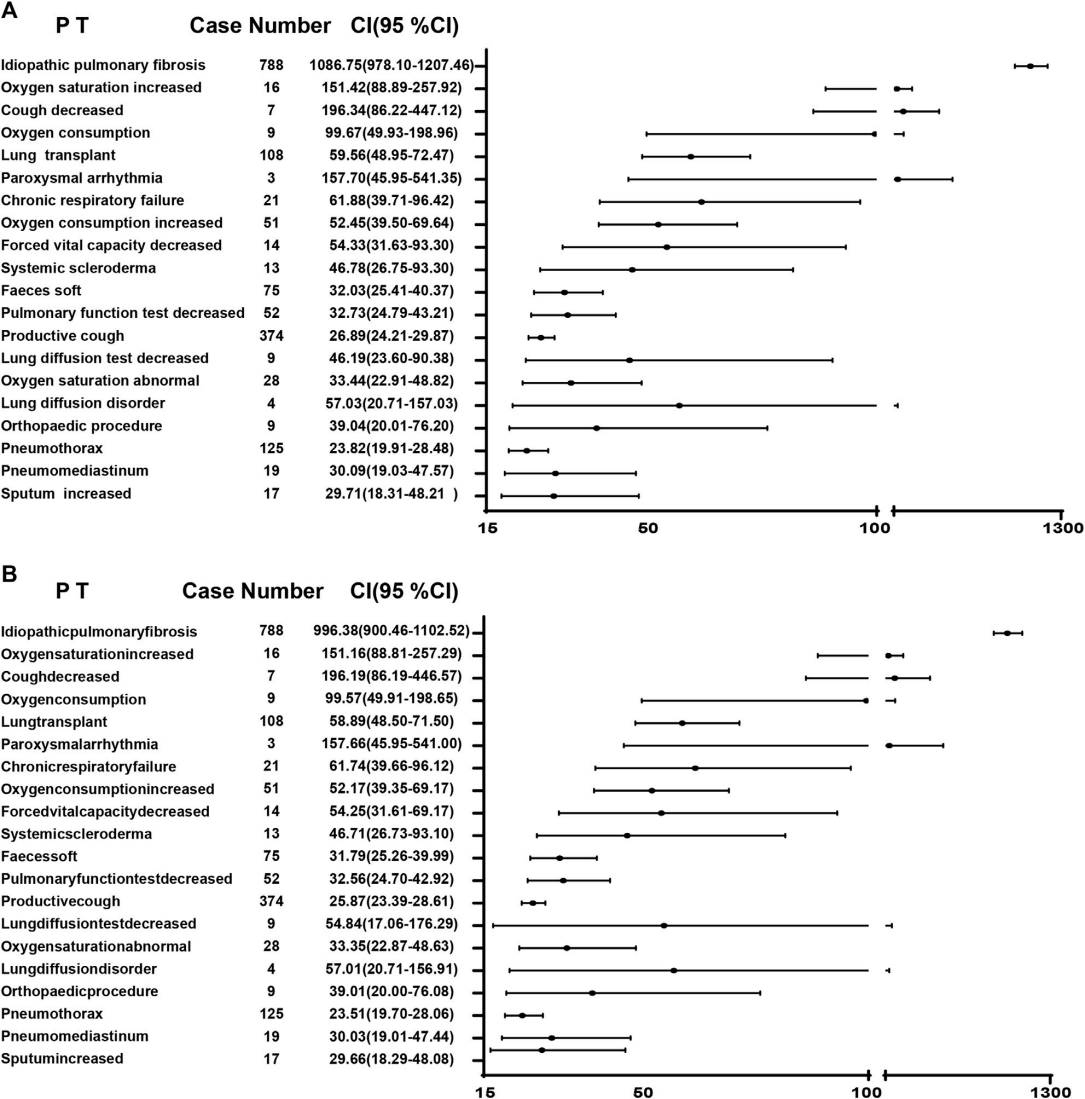
The ROR **(A)** and PRR **(B)** disproportionality analysis of signals at PTs level associated with nintedanib in the FAERS database. ROR, reporting odds ratio; PRR, proportional reporting ratio; PTs, preferred terms; FAERS, Food and Drug Administration Adverse Event Reporting System; 95% CI, 95% credibility interval.

A combination of PRR and ROR algorithms was utilized to analyze AEs and assess their adherence to various screening criteria. In the FAERS database, a total of 150 PTs (Supplementary Table S1) related to pirfenidone was screened, and the top 20 PTs, presented in Figure 2, are ranked by their lower 95% CI values. The top five robust PTs were idiopathic pulmonary fibrosis (lower 95% CI: 52.21), lung diffusion test decreased (lower 95% CI: 48.29), forced vital capacity decreased (lower 95% CI: 36.64), forced vital capacity abnormal (lower 95% CI: 35.99), and sunburn (lower 95% CI: 30.91). For nintedanib, 422 PTs (Supplementary Table S2) were identified, and the top five PTs were idiopathic pulmonary fibrosis (lower 95% CI: 978.10), oxygen saturation increased (lower 95% CI: 88.89), cough decreased (lower 95% CI: 86.22), oxygen consumption (lower 95% CI: 49.93), and lung transplant (lower 95% CI: 48.95) (Figure 3). In the VigiAccess database, the top 20 PTs were ranked by case numbers. The top five PTs for pirfenidone were death (case number: 6,240), nausea (case numbers: 5,013), fatigue (case number: 3,760), decreased appetite (case number: 3,733), and diarrhoea (case number: 2,895) (Table 1). The top five PTs for nintedanib were diarrhoea (case numbers: 9,676), nausea (case number: 3,770), weight decreased (case number: 2,888), decreased appetite (case number: 2,671), and vomiting (case number: 2,302) (Table 1).

**TABLE 1:** The signals of AEs of pirfenidone and nintedanib at the PT level in the VigiAccess database.

PT name	Case numbers	PT name	Case numbers
Death	6,240	Diarrhoea	9,676
Nausea	5,013	Nausea	3,770
Fatigue	3,760	Weight decreased	2,888
Decreased appetite	3,733	Decreased appetite	2,671
Diarrhoea	2,895	Vomiting	2,302
Dyspnoea	2,527	Dyspnoea	2,243
Weight decreased	2,455	Fatigue	2078
Rash	2,345	Cough	1,536
Dizziness	2004	Abdominal pain upper	1,426
Cough	1751	Death	1,424
Abdominal discomfort	1,602	ldopathic pulmonary fbrosis	1,326
Pneumonia	1,586	Asthenia	1,162
Vomiting	1,566	Constipation	1,139
Pruritus	1,398	Headache	1,075
Asthenia	1,376	Abdominal discomfort	1,056
Abdominal pain upper	1,329	Abdominal pain	1,035
Malaise	1,321	Pneumonia	971
Dyspepsia	1,271	Dizziness	813
Headache	1,246	Productive cough	728
Photosensitivity reaction	1,194	Flatulence	708

Abbreviations: AEs, adverse events; PT, preferred term.

### 3.3 Signal detection at SOC level

In the FAERS database, gastrointestinal disorders ranked first among 19 SOCs for pirfenidone (case number: 9,927; 30.63%), and also ranked first among 23 SOCs for nintedanib (case number: 8,988; 35.88%) (Tables 2, 3 Supplementary Table S3). Additionally, in the VigiAccess database, gastrointestinal disorders ranked second among 27 SOCs for pirfenidone (case number: 11,442, 15.75%), following general disorders and administration site conditions (case number: 15,378, 21.17%) (Table 4). For nintedanib, gastrointestinal disorders remained the top SOC (case number: 13,433, 22.77%), followed by general disorders and administration site conditions (case number: 7,425, 12.59%) (Table 5).

**TABLE 2:** The signals of AEs of pirfenidone at the SOC level in the FEARS database.

SOC name	Case number	PT	Percentage (%)
Gastrointestinal disorders	9,927	21	30.63
General disorders and administration site conditions	8,009	8	24.71
Respiratory, thoracic and mediastinal disorders	4,694	44	14.48
Metabolism and nutrition disorders	2,168	7	6.69
Skin and subcutaneous tissue disorders	1969	9	6.08
Investigations	1925	21	5.94
Cardiac disorders	1,343	6	4.14
Psychiatric disorders	759	5	2.34
Injury, poisoning and procedural complications	748	6	2.31
Nervous system disorders	406	4	1.25
Immune system disorders	228	2	0.70
Renal and urinary disorders	69	1	0.21
Vascular disorders	66	5	0.20
Surgical and medical procedures	66	5	0.20
Social circumstances	14	1	0.04
Musculoskeletal and connective tissue disorders	8	1	0.02
Reproductive system and breast disorders	6	2	0.02
Infections and infestations	3	1	0.01
Ear and labyrinth disorders	3	1	0.01

Abbreviations: AEs, adverse events; SOC, system organ class; FAERS, food and drug administration adverse event reporting system; PT, preferred term.

**TABLE 3:** The signals of AEs of nintedanib at the SOC level in the FEARS database.

SOC name	Case number	PT	Percentage (%)
Gastrointestinal disorders	85	8,988	35.88
Respiratory, thoracic and mediastinal disorders	65	4,384	17.50
General disorders and administration site conditions	18	2,712	10.83
Investigations	49	1987	7.93
Metabolism and nutrition disorders	14	1,467	5.86
Immune system disorders	4	950	3.79
Vascular disorders	28	891	3.56
Cardiac disorders	22	759	3.03
Nervous system disorders	16	548	2.19
Hepatobiliary disorders	19	465	1.86
Injury, poisoning and procedural complications	14	455	1.82
Surgical and medical procedures	24	322	1.29
Renal and urinary disorders	13	243	0.97
Infections and infestations	14	224	0.89
Neoplasms benign, malignant and unspecified (incl cysts and polyps)	8	200	0.80
Musculoskeletal and connective tissue disorders	6	201	0.80
Psychiatric disorders	5	100	0.40
Blood and lymphatic system disorders	6	66	0.26
Skin and subcutaneous tissue disorders	4	38	0.15
Eye disorders	2	26	0.10
Reproductive system and breast disorders	3	12	0.05
Social circumstances	2	9	0.04
Ear and labyrinth disorders	1	6	0.02

Abbreviations: AEs, adverse events; SOC, system organ class; FAERS, food and drug administration adverse event reporting system; PT, preferred term.

**TABLE 4:** The signals of AEs of pirfenidone at the SOC level in the VigiAccess database.

SOC name	Case number	PT	Percentage (%)
General disorders and administration site conditions	15378	146	21.17
Gastrointestinal disorders	11442	246	15.75
Respiratory, thoracic and mediastinal disorders	6,260	196	8.62
Skin and subcutaneous tissue disorders	5,773	158	7.95
Nervous system disorders	5,511	161	7.59
Investigations	5,012	304	6.90
Metabolism and nutrition disorders	4,362	63	6.01
Infections and infestations	4,177	222	5.75
Injury, poisoning and procedural complications	3,513	190	4.84
Psychiatric disorders	2,366	107	3.26
Musculoskeletal and connective tissue disorders	1769	112	2.44
Cardiac disorders	1,427	103	1.96
Vascular disorders	1,048	75	1.44
Neoplasms benign, malignant and unspecified (incl cysts and polyps)	796	198	1.10
Surgical and medical procedures	671	99	0.92
Renal and urinary disorders	660	67	0.91
Eye disorders	591	75	0.81
Hepatobiliary disorders	447	51	0.62
Ear and labyrinth disorders	346	26	0.48
Blood and lymphatic system disorders	331	49	0.46
Immune system disorders	328	23	0.45
Social circumstances	158	27	0.22
Reproductive system and breast disorders	109	49	0.15
Product issues	79	30	0.11
Endocrine disorders	51	19	0.07
Congenital, familial and genetic disorders	18	11	0.02
Pregnancy, puerperium and perinatal conditions	3	2	0.00

Abbreviations: AEs, adverse events; SOC, system organ class; PT, preferred term.

**TABLE 5:** The signals of AEs of nintedanib at the SOC level in the VigiAccess database.

SOC name	Case number	PT	Percentage (%)
Gastrointestinal disorders	13433	303	22.77
General disorders and administration site conditions	7,425	172	12.59
Respiratory, thoracic and mediastinal disorders	6,388	178	10.83
Investigations	6,084	361	10.31
Nervous system disorders	3,670	198	6.22
Metabolism and nutrition disorders	3,577	84	6.06
Infections and infestations	3,474	275	5.89
Musculoskeletal and connective tissue disorders	1908	125	3.23
Injury, poisoning and procedural complications	1886	217	3.20
Skin and subcutaneous tissue disorders	1,501	136	2.54
Psychiatric disorders	1,375	114	2.33
Cardiac disorders	1,363	101	2.31
Vascular disorders	1,360	94	2.31
Hepatobiliary disorders	1,002	58	1.70
Neoplasms benign, malignant and unspecified (incl cysts and polyps)	995	170	1.69
Renal and urinary disorders	824	82	1.40
Surgical and medical procedures	734	174	1.24
Blood and lymphatic system disorders	694	65	1.18
Eye disorders	445	76	0.75
Immune system disorders	230	25	0.39
Ear and labyrinth disorders	220	26	0.37
Reproductive system and breast disorders	134	49	0.23
Social circumstances	130	32	0.22
Product issues	58	26	0.10
Endocrine disorders	48	11	0.08
Congenital, familial and genetic disorders	22	18	0.04
Pregnancy, puerperium and perinatal conditions	3	3	0.01

Abbreviations: AEs, adverse events; SOC, system organ class; PT, preferred term.

## 4 Discussion

Pirfenidone and nintedanib are major therapeutic agents for IPF patients, having demonstrated their efficacy and safety through rigorous clinical trials (Justet et al., 2023). However, there’s a limited number of post-marketing and pharmacovigilance studies that offer insights into their long-term safety profiles and comprehensive guidelines. Given that “a one-size-fits-all approach” to the management of IPF is not currently suitable, the drugs should be decided with optimal consideration of interindividual differences to maximize effectives and minimize side-effects (Karampitsakos et al., 2023a). In this study, we analyzed and compared the AEs induced by these two drugs using data from the FAERS and VigiAccess databases in the real-world practice. This analysis aims to assist clinicians in managing these drugs more effectively for IPF patients.

In our analysis, we found that male patients or those over 65 accounted for a large part of AE reports in both databases. This is consistent with previous studies, indicating that older age and male gender are risk factors for IPF (Zhao et al., 2023). The higher incidence in males might be due to factors like smoking or occupational exposures. Hence, it is crucial for clinicians to be particularly attentive when administering pirfenidone or nintedanib to elderly males, given their heightened susceptibility to AEs. Moreover, hospitalization was the most severe outcome for both pirfenidone (10,869, 19.43%) and (nintedanib) (17,433, 48.58%). In 10%–20% of hospitalized patients, ADRs pose unexpected risks to patients, affecting their prognosis and increasing their financial burden (Staniszewka, 2020). In this study, therefore, we aim to identify the ADRs of these two drugs and offer guidance on their prevention or control, especially for the new and unexpected ADRs.

Both pirfenidone and nintedanib have been linked to several ADRs. Pirfenidone is mainly associated with gastrointestinal disorders, skin rashes or photosensitivity, while nintedanib is tied to gastrointestinal disorders, bleeding, cardiovascular events and myocardial infarction (Petnak et al., 2023; Rogliani et al., 2023). For pirfenidone in the FAERS database, the top five signals at PT level are idiopathic pulmonary fibrosis, lung diffusion test decreased, forced vital capacity decreased, forced vital capacity abnormal, and sunburn. Other uncommon signals included chromaturia, blood bilirubin decreased, coronary artery arteriosclerosis, allergic sinusitis and liver function increased/decreased. This highlights the importance of conventional monitoring for patients taking pirfenidone. And if respiratory symptoms worsen with the use of pirfenidone over time, temporary dosage reductions or discontinuations may be required (Meyer and Decker, 2023). Additionally, daily use of sunscreen and protective clothing to avoid Sun exposure and sunlamps should be recommended. For nintedanib, the top five are idiopathic pulmonary fibrosis, oxygen saturation increased, cough decreased, oxygen consumption, lung transplant, which also indicates that professional caregivers should pay more attention to the pulmonary protection. Other uncommon signals are paroxysmal arrhythmia, cholelithotomy, aortic valve calcification, haemorrhoids, and hepatic cirrhosis, cystitis, reminding us that we should not only focus on respiratory system but also pay attention to cardiovascular and digestive systems in the administration of nintedanib. In the VigiAccess database, gastrointestinal disorders, including nausea, vomiting and diarrhoea, account for majority ADRs of reports, which are consistent with clinical trials (Song et al., 2019). Hence, Supportive care, including antiemetic or antidiarrheal therapy, must be prepared for patients with high risk potential of gastrointestinal disorders. Additionally, more attention should be paid to signals that were rarely mentioned in these drugs labels. Hemiplegic migraine occurred with the use of pirfenidone, and asthenia, constipation, as well as flatulence occurred with the use of nintedanib. Among these, only asthenia has been reported in patients with advanced solid tumors or lung adenocarcinoma (Bahleda et al., 2015; Riudavets et al., 2021). Recent studies found that IPF patients exhibit an approximately five-fold elevated susceptibility to developing lung cancer compared with the general population, and those with coexisting IPF and lung cancer experience a markedly diminished overall survival rate compared with IPF patients without lung cancer (Karampitsakos et al., 2023b; Karampitsakos et al., 2023c). Therefore, the role of antifibrotics compounds plus antitumor drugs in IPF patients with lung cancer should be explored. In a clinical trial, chemotherapy, carboplatin plus nab-paclitaxel, has demonstrated efficacy and tolerability in advanced NSCLC patients with IPF, and the combination of nintedanib with this chemotherapy improved overall survival (Otsubo et al., 2022). In another study, pembrolizumab, the PD-1 inhibitor, was found to exert therapeutic effect in bleomycin-induced pulmonary fibrosis (Karampitsakos et al., 2023d). More clinical trials are imperative to find the potential of ADRs in the use of antifibrotic and antitumor medications. The high prevalence of depression in IPF patients receiving antifibrotic drugs, which reduce the quality of life, should also be noted (Tzouvelekis et al., 2020). Furthermore, in combined use of pirfenidone and nintedanib, no relevant pharmacokinetic drug-drug interaction was found, which needs further study to assess the efficiveness and safety of this combination compared to monotherapy in IPF patients (Richeldi et al., 2019).

## 5 Limitations

It is important to acknowledge the limitations in this study. First, the major is the issue of underreporting of AEs by healthcare professionals, leading to incomplete data and an inaccurate representation of the true safety profile of drug. Second, the quality of data in pharmacovigilance databases can exhibit variations that hinder the analysis and interpretation of safety signals. Third, pharmacovigilance data often lacks comprehensive information about confounding factors that could contribute to AEs. Therefore, it is important to consider these limitations when interpreting pharmacovigilance data and making decisions regarding drug and risk management. However, this study succeeded in identifying previous unknown or underestimated risk associated with pirfenidone and nintedanib, including signals like hemiplegic migraine, asthenia, constipation, and flatulence. These findings offer an opportunity to assess ADRs that may remain undetected during pre-approval clinical trials, which typically involve a limited number of patients.

## 6 Conclusion

Our analysis provides valuable insights into the safety profiles of pirfenidone and nintedanib in real-world practice. The findings emphasize the importance of monitoring and managing specific ADRs, highlighting the ongoing need for pharmacovigilance efforts to improve patient safety, medical decision-making and effectiveness of drug therapy.

## Data Availability

The original contributions presented in the study are included in the article/Supplementary Material, further inquiries can be directed to the corresponding authors.
