# Supplementary material for: A real-world study of antifibrotic drugs-related adverse events based on the United States food and drug administration adverse event reporting system and VigiAccess databases
Source: Front Pharmacol. 2024 Feb 23;15:1310286. doi: 10.3389/fphar.2024.1310286 (PMC10920264; doi:10.3389/fphar.2024.1310286)
Supplement: Supplementary file 1 [file Table1.docx]

**Supplemental Materials**

Table S1 The signals of pirfenidone at the PT level in the FAERS database

| **PTs** | **Case Number** | **ROR** | **95%CI** | **PRR** | | **95%CI** | |
| --- | --- | --- | --- | --- | --- | --- | --- |
| idiopathic pulmonary fibrosis | 225 | 60.55 | 52.51-69.82 | 60.01 | | 52.1-69.13 | |
| carbon monoxide diffusing capacity decreased | 12 | 98.02 | 51.51-186.53 | 97.97 | | 51.5-186.4 | |
| forced vital capacity decreased | 33 | 55.86 | 38.64-80.77 | 55.79 | | 38.6-80.63 | |
| lung diffusion test decreased | 19 | 60.62 | 37.18-98.83 | 60.57 | | 37.16-98.72 | |
| forced vital capacity abnormal | 4 | 111.60 | 35.99-346.05 | 111.58 | | 35.99-345.95 | |
| sunburn | 224 | 35.50 | 30.91-40.76 | 35.19 | | 30.68-40.36 | |
| photosensitivity reaction | 447 | 28.85 | 26.18-31.81 | 28.36 | | 25.77-31.2 | |
| dependence on oxygen therapy | 14 | 37.21 | 21.42-64.65 | 37.19 | | 21.42-64.6 | |
| pulmonary function test abnormal | 45 | 28.52 | 21.03-38.68 | 28.48 | | 21.01-38.6 | |
| solar dermatitis | 9 | 33.86 | 17.06-67.22 | 33.85 | | 17.06-67.18 | |
| pulmonary function test decreased | 75 | 18.36 | 14.55-23.18 | 18.31 | | 14.52-23.1 | |
| total lung capacity decreased | 24 | 19.61 | 12.99-29.61 | 19.59 | | 12.99-29.57 | |
| sputum increased | 26 | 17.71 | 11.93-26.27 | 17.69 | | 11.93-26.24 | |
| fev1/fvc ratio decreased | 4 | 28.49 | 10.26-79.09 | 28.49 | | 10.26-79.07 | |
| decreased appetite | 1838 | 9.43 | 8.98-9.89 | 8.80 | | 8.42-9.21 | |
| fibrosis | 30 | 11.93 | 8.28-17.17 | 11.91 | | 8.28-17.14 | |
| pulmonary fibrosis | 135 | 9.50 | 8-11.28 | 9.46 | | 7.97-11.21 | |
| heavy exposure to ultraviolet light | 4 | 21.60 | 7.86-59.37 | 21.60 | | 7.86-59.36 | |
| hemiplegic migraine | 5 | 17.62 | 7.17-43.32 | 17.62 | | 7.17-43.3 | |
| pulmonary artery dilatation | 5 | 17.26 | 7.02-42.4 | 17.25 | | 7.02-42.39 | |
| dyspepsia | 657 | 7.56 | 6.99-8.17 | 7.38 | | 6.84-7.97 | |
| productive cough | 274 | 7.34 | 6.5-8.27 | 7.27 | | 6.45-8.19 | |
| gastric disorder | 359 | 7.18 | 6.46-7.97 | 7.09 | | 6.39-7.86 | |
| restrictive pulmonary disease | 10 | 11.43 | 6.08-21.47 | 11.42 | | 6.08-21.45 | |
| lower respiratory tract congestion | 16 | 9.89 | 6.01-16.26 | 9.88 | | 6.01-16.24 | |
| oxygen consumption increased | 23 | 8.94 | 5.91-13.53 | 8.93 | | 5.9-13.51 | |
| lung transplant | 40 | 7.89 | 5.76-10.79 | 7.88 | | 5.76-10.77 | |
| food aversion | 12 | 10.12 | 5.7-17.98 | 10.12 | | 5.7-17.97 | |
| asbestosis | 3 | 17.62 | 5.52-56.27 | 17.62 | | 5.52-56.25 | |
| oxygen saturation decreased | 243 | 6.02 | 5.3-6.84 | 5.97 | | 5.26-6.77 | |
| abdominal discomfort | 992 | 5.64 | 5.29-6.02 | 5.46 | | 5.13-5.81 | |
| lung diffusion disorder | 3 | 16.74 | 5.25-53.38 | 16.74 | | 5.25-53.36 | |
| death | 4830 | 5.09 | 4.93-5.25 | 4.29 | | 4.19-4.41 | |
| gastrooesophageal reflux disease | 359 | 5.41 | 4.87-6.01 | 5.34 | | 4.82-5.93 | |
| ageusia | 120 | 5.68 | 4.74-6.81 | 5.66 | | 4.73-6.77 | |
| weight decreased | 1155 | 5.01 | 4.72-5.31 | 4.82 | | 4.55-5.1 | |
| small cell lung cancer | 13 | 8.15 | 4.7-14.14 | 8.15 | | 4.7-14.13 | |
| hypogeusia | 13 | 8.11 | 4.68-14.06 | 8.10 | | 4.68-14.05 | |
| pulmonary pain | 20 | 7.27 | 4.67-11.33 | 7.27 | | 4.67-11.32 | |
| idiopathic pneumonia syndrome | 3 | 14.15 | 4.46-44.91 | 14.14 | | 4.46-44.9 | |
| product dose omission in error | 95 | 5.44 | 4.44-6.67 | 5.42 | | 4.43-6.64 | |
| paranasal sinus hypersecretion | 21 | 6.77 | 4.39-10.43 | 6.77 | | 4.39-10.42 | |
| nausea | 3135 | 4.53 | 4.36-4.7 | 4.08 | | 3.95-4.22 | |
| international normalised ratio decreased | 18 | 6.95 | 4.35-11.08 | 6.94 | | 4.35-11.07 | |
| abdominal pain upper | 892 | 4.65 | 4.35-4.98 | 4.52 | | 4.24-4.83 | |
| sputum abnormal | 6 | 9.75 | 4.33-21.96 | 9.75 | | 4.33-21.95 | |
| pneumothorax | 76 | 5.36 | 4.27-6.73 | 5.35 | | 4.27-6.71 | |
| chronic respiratory failure | 8 | 8.40 | 4.16-16.94 | 8.39 | | 4.16-16.93 | |
| clubbing | 4 | 11.07 | 4.09-29.97 | 11.07 | | 4.09-29.96 | |
| intentional product use issue | 416 | 4.38 | 3.98-4.83 | 4.33 | | 3.93-4.76 | |
| increased viscosity of bronchial secretion | 4 | 10.22 | 3.78-27.65 | 10.22 | | 3.78-27.64 | |
| no adverse event | 816 | 3.78 | 3.53-4.06 | 3.69 | | 3.45-3.95 | |
| oral discharge | 3 | 11.04 | 3.49-34.86 | 11.04 | | 3.49-34.85 | |
| oxygen therapy | 10 | 6.28 | 3.36-11.75 | 6.28 | | 3.36-11.74 | |
| cough | 883 | 3.49 | 3.27-3.74 | 3.40 | | 3.19-3.63 | |
| eructation | 61 | 4.20 | 3.26-5.41 | 4.19 | | 3.26-5.4 | |
| penile discharge | 3 | 10.25 | 3.25-32.33 | 10.25 | | 3.25-32.32 | |
| lung disorder | 171 | 3.73 | 3.2-4.34 | 3.71 | | 3.19-4.31 | |
| oesophageal pain | 16 | 5.15 | 3.14-8.44 | 5.15 | | 3.14-8.44 | |
| catarrh | 5 | 7.54 | 3.11-18.3 | 7.54 | | 3.11-18.29 | |
| pneumonia | 896 | 3.26 | 3.05-3.49 | 3.18 | | 2.98-3.39 | |
| diarrhoea | 1865 | 3.11 | 2.96-3.26 | 2.95 | | 2.82-3.08 | |
| taste disorder | 64 | 3.68 | 2.88-4.71 | 3.67 | | 2.87-4.7 | |
| rash | 1236 | 3.03 | 2.87-3.21 | 2.93 | | 2.78-3.1 | |
| dysgeusia | 209 | 3.24 | 2.82-3.71 | 3.22 | | 2.81-3.69 | |
| fatigue | 2148 | 2.88 | 2.76-3.02 | 2.72 | | 2.61-2.83 | |
| feeding disorder | 67 | 3.47 | 2.73-4.41 | 3.46 | | 2.72-4.4 | |
| pulmonary hypertension | 71 | 3.42 | 2.7-4.32 | 3.41 | | 2.7-4.31 | |
| chromaturia | 69 | 3.34 | 2.63-4.23 | 3.33 | | 2.63-4.22 | |
| blood bilirubin decreased | 4 | 7.05 | 2.62-18.97 | 7.05 | | 2.62-18.97 | |
| flatulence | 145 | 2.99 | 2.54-3.52 | 2.98 | | 2.53-3.51 | |
| dyspnoea exertional | 95 | 3.07 | 2.51-3.76 | 3.06 | | 2.5-3.74 | |
| lung infection | 54 | 3.22 | 2.46-4.21 | 3.22 | | 2.46-4.2 | |
| ill-defined disorder | 132 | 2.89 | 2.44-3.44 | 2.88 | | 2.43-3.42 | |
| defaecation disorder | 4 | 6.44 | 2.39-17.32 | 6.44 | | 2.39-17.31 | |
| pulmonary thrombosis | 36 | 3.30 | 2.38-4.59 | 3.30 | | 2.38-4.58 | |
| gastric infection | 14 | 4.00 | 2.36-6.78 | 4.00 | | 2.36-6.77 | |
| faeces soft | 22 | 3.58 | 2.35-5.44 | 3.57 | | 2.35-5.44 | |
| acute respiratory failure | 51 | 3.08 | 2.34-4.06 | 3.07 | | 2.33-4.05 | |
| dyspnoea | 1267 | 2.46 | 2.33-2.61 | 2.39 | | 2.26-2.52 | |
| dizziness | 1182 | 2.47 | 2.33-2.61 | 2.40 | | 2.27-2.53 | |
| bronchiectasis | 20 | 3.61 | 2.32-5.61 | 3.61 | | 2.32-5.6 | |
| epigastric discomfort | 15 | 3.86 | 2.32-6.42 | 3.86 | | 2.32-6.42 | |
| bronchial carcinoma | 4 | 6.23 | 2.32-16.75 | 6.23 | | 2.32-16.74 | |
| upper-airway cough syndrome | 27 | 3.38 | 2.31-4.94 | 3.38 | | 2.31-4.93 | |
| investigation abnormal | 6 | 5.12 | 2.29-11.48 | 5.12 | | 2.29-11.47 | |
| hiatus hernia | 31 | 3.26 | 2.29-4.64 | 3.25 | | 2.29-4.63 | |
| pharyngeal mass | 3 | 7.07 | 2.25-22.19 | 7.07 | | 2.25-22.19 | |
| chapped lips | 14 | 3.79 | 2.24-6.42 | 3.79 | | 2.24-6.42 | |
| rhinorrhoea | 163 | 2.58 | 2.21-3.02 | 2.57 | | 2.21-3 | |
| rash pruritic | 119 | 2.64 | 2.2-3.16 | 2.63 | | 2.19-3.15 | |
| gastrointestinal disorder | 205 | 2.52 | 2.19-2.89 | 2.50 | | 2.18-2.87 | |
| thermal burn | 22 | 3.31 | 2.18-5.04 | 3.31 | | 2.18-5.04 | |
| respiratory disorder | 73 | 2.74 | 2.17-3.45 | 2.73 | | 2.17-3.44 | |
| vomiting | 976 | 2.30 | 2.16-2.46 | 2.25 | | 2.12-2.4 | |
| burns third degree | 5 | 5.20 | 2.15-12.58 | 5.20 | | 2.15-12.57 | |
| Insomnia | 598 | 2.33 | 2.15-2.53 | 2.30 | | 2.12-2.49 | |
| Hypophagia | 57 | 2.77 | 2.13-3.59 | 2.76 | | 2.13-3.58 | |
| skin reaction | 41 | 2.89 | 2.12-3.93 | 2.89 | | 2.12-3.92 | |
| eating disorder | 58 | 2.74 | 2.12-3.55 | 2.74 | | 2.11-3.54 | |
| gastric ph decreased | 5 | 5.06 | 2.09-12.23 | 5.06 | | 2.09-12.23 | |
| liver function test increased | 67 | 2.63 | 2.07-3.35 | 2.63 | | 2.07-3.34 | |
| blood sodium decreased | 40 | 2.81 | 2.05-3.83 | 2.80 | | 2.05-3.82 | |
| lung neoplasm | 9 | 3.94 | 2.04-7.61 | 3.94 | | 2.04-7.61 | |
| Parosmia | 20 | 3.15 | 2.03-4.9 | 3.15 | | 2.03-4.89 | |
| liver function test decreased | 3 | 6.36 | 2.03-19.92 | 6.36 | | 2.03-19.92 | |
| Anosmia | 26 | 2.93 | 1.99-4.31 | 2.93 | | 1.99-4.3 | |
| exercise tolerance decreased | 17 | 3.17 | 1.97-5.11 | 3.17 | | 1.97-5.11 | |
| lower respiratory tract infection | 97 | 2.38 | 1.95-2.91 | 2.38 | | 1.95-2.9 | |
| Sneezing | 52 | 2.56 | 1.95-3.36 | 2.55 | | 1.95-3.36 | |
| Hypersomnia | 67 | 2.47 | 1.94-3.14 | 2.47 | | 1.94-3.14 | |
| arteriosclerosis coronary artery | 14 | 3.28 | 1.94-5.55 | 3.28 | | 1.94-5.55 | |
| Haemoptysis | 60 | 2.41 | 1.87-3.11 | 2.41 | | 1.87-3.1 | |
| respiratory failure | 138 | 2.19 | 1.85-2.59 | 2.18 | | 1.85-2.58 | |
| aortic arteriosclerosis | 6 | 4.11 | 1.84-9.19 | 4.11 | | 1.84-9.19 | |
| hepatic enzyme increased | 127 | 2.16 | 1.82-2.58 | 2.16 | | 1.81-2.57 | |
| limb mass | 8 | 3.60 | 1.79-7.22 | 3.59 | | 1.79-7.21 | |
| arterial occlusive disease | 16 | 2.91 | 1.78-4.77 | 2.91 | | 1.78-4.76 | |
| oxygen saturation abnormal | 8 | 3.56 | 1.77-7.15 | 3.56 | | 1.77-7.15 | |
| appetite disorder | 18 | 2.82 | 1.77-4.48 | 2.82 | | 1.77-4.48 | |
| sputum discoloured | 21 | 2.71 | 1.76-4.16 | 2.71 | | 1.76-4.16 | |
| abdominal distension | 184 | 2.03 | 1.75-2.35 | 2.02 | | 1.75-2.33 | |
| Rales | 13 | 3.03 | 1.75-5.23 | 3.03 | | 1.75-5.22 | |
| rash erythematous | 71 | 2.16 | 1.71-2.73 | 2.16 | | 1.71-2.73 | |
| upper respiratory tract congestion | 8 | 3.37 | 1.68-6.76 | 3.37 | | 1.68-6.76 | |
| painful respiration | 7 | 3.51 | 1.67-7.4 | 3.51 | | 1.67-7.4 | |
| pulmonary oedema | 79 | 2.04 | 1.63-2.54 | 2.03 | | 1.63-2.53 | |
| pharyngeal disorder | 9 | 3.08 | 1.6-5.93 | 3.08 | | 1.6-5.93 | |
| nasal crusting | 3 | 4.95 | 1.58-15.47 | 4.95 | | 1.58-15.47 | |
| excessive cerumen production | 3 | 4.81 | 1.54-15.02 | 4.80 | | 1.54-15.02 | |
| Mass | 30 | 2.18 | 1.52-3.12 | 2.18 | | 1.52-3.12 | |
| Hyperaesthesia | 19 | 2.38 | 1.52-3.75 | 2.38 | | 1.52-3.74 | |
| oesophageal achalasia | 3 | 4.59 | 1.47-14.33 | 4.59 | | 1.47-14.33 | |
| chest injury | 6 | 3.22 | 1.44-7.21 | 3.22 | | 1.44-7.2 | |
| sinus congestion | 22 | 2.15 | 1.41-3.26 | 2.14 | | 1.41-3.26 | |
| faeces pale | 6 | 3.12 | 1.4-6.97 | 3.12 | | 1.4-6.97 | |
| Emphysema | 18 | 2.19 | 1.38-3.49 | 2.19 | | 1.38-3.48 | |
| pulmonary mycosis | 3 | 4.27 | 1.37-13.35 | 4.27 | | 1.37-13.34 | |
| aortic dilatation | 3 | 4.12 | 1.32-12.85 | 4.12 | | 1.32-12.85 | |
| carbon dioxide increased | 4 | 3.51 | 1.31-9.39 | 3.51 | | 1.31-9.39 | |
| allergic sinusitis | 3 | 3.91 | 1.25-12.2 | 3.91 | | 1.25-12.19 | |
| irregular breathing | 3 | 3.91 | 1.25-12.2 | 3.91 | | 1.25-12.19 | |
| sinus operation | 6 | 2.79 | 1.25-6.24 | 2.79 | | 1.25-6.24 | |
| increased upper airway secretion | 7 | 2.59 | 1.23-5.45 | 2.59 | | 1.23-5.45 | |
| initial insomnia | 15 | 2.03 | 1.22-3.37 | 2.03 | | 1.22-3.37 | |
| prostate infection | 3 | 3.75 | 1.2-11.69 | 3.75 | | 1.2-11.69 | |
| cardiac fibrillation | 4 | 3.09 | 1.15-8.26 | 3.09 | | 1.15-8.26 | |
| nasal disorder | 7 | 2.30 | 1.09-4.83 | 2.30 | | 1.09-4.83 | |
| subcutaneous emphysema | 3 | 3.30 | 1.06-10.3 | 3.30 | | 1.06-10.3 | |
| gallbladder operation | 7 | 2.22 | 1.06-4.67 | 2.22 | | 1.06-4.67 | |
| prostatic operation | 3 | 3.27 | 1.05-10.2 | 3.27 | | 1.05-10.2 | |
| listless | 6 | 2.32 | 1.04-5.18 | 2.32 | | 1.04-5.18 | |
| FAERS, Food and Drug Administration Adverse Event Reporting System; PTs, preferred terms; ROR, reporting odds ratio; 95% CI, 95% credibility interval. | | | | |  | |  |

| Table S2 The signals of nintedanib at the PT level in the FAERS database | | | | | |  |
| --- | --- | --- | --- | --- | --- | --- |
| **PTs** | **Case Number** | **ROR** | **95%CI** | **PRR** | **95%CI** | |
| idiopathic pulmonary fibrosis | 788 | 1086.74 | 978.1-1207.46 | 996.38 | 900.46-1102.52 | |
| oxygen saturation increased | 16 | 151.42 | 88.89-257.92 | 151.16 | 88.81-257.29 | |
| cough decreased | 7 | 196.34 | 86.22-447.12 | 196.2 | 86.2-446.57 | |
| oxygen consumption | 9 | 99.67 | 49.93-198.96 | 99.57 | 49.91-198.65 | |
| lung transplant | 108 | 59.56 | 48.95-72.47 | 58.89 | 48.5-71.5 | |
| paroxysmal arrhythmia | 3 | 157.71 | 45.95-541.35 | 157.66 | 45.95-541 | |
| chronic respiratory failure | 21 | 61.88 | 39.71-96.42 | 61.74 | 39.66-96.12 | |
| oxygen consumption increased | 51 | 52.45 | 39.5-69.64 | 52.17 | 39.35-69.17 | |
| forced vital capacity decreased | 14 | 54.33 | 31.63-93.3 | 54.25 | 31.61-93.1 | |
| systemic scleroderma | 13 | 46.78 | 26.75-81.8 | 46.71 | 26.73-81.63 | |
| faeces soft | 75 | 32.03 | 25.41-40.37 | 31.79 | 25.26-39.99 | |
| pulmonary function test decreased | 52 | 32.73 | 24.79-43.21 | 32.56 | 24.7-42.92 | |
| productive cough | 374 | 26.89 | 24.21-29.87 | 25.87 | 23.39-28.61 | |
| oxygen saturation abnormal | 28 | 33.44 | 22.91-48.82 | 33.35 | 22.87-48.63 | |
| lung diffusion disorder | 4 | 57.03 | 20.71-157.03 | 57.01 | 20.71-156.91 | |
| orthopaedic procedure | 9 | 39.04 | 20.01-76.2 | 39.01 | 20-76.08 | |
| pneumothorax | 125 | 23.82 | 19.91-28.48 | 23.51 | 19.71-28.06 | |
| pneumomediastinum | 19 | 30.09 | 19.03-47.57 | 30.03 | 19.01-47.44 | |
| lung diffusion test decreased | 6 | 42.78 | 18.83-97.19 | 42.76 | 18.83-97.08 | |
| sputum increased | 17 | 29.71 | 18.31-48.21 | 29.66 | 18.29-48.08 | |
| abnormal loss of weight | 51 | 23.72 | 17.95-31.36 | 23.6 | 17.88-31.15 | |
| dyspnoea exertional | 233 | 20.08 | 17.61-22.91 | 19.61 | 17.25-22.3 | |
| carbon monoxide diffusing capacity decreased | 3 | 54.86 | 17.06-176.41 | 54.84 | 17.06-176.29 | |
| food aversion | 13 | 29.27 | 16.83-50.9 | 29.23 | 16.82-50.79 | |
| diverticulitis intestinal haemorrhagic | 5 | 39.31 | 16.03-96.4 | 39.29 | 16.03-96.31 | |
| diarrhoea | 2808 | 16.23 | 15.53-16.97 | 11.72 | 11.35-12.09 | |
| total lung capacity decreased | 12 | 25.58 | 14.4-45.44 | 25.54 | 14.39-45.35 | |
| large intestinal polypectomy | 3 | 45.06 | 14.1-143.98 | 45.05 | 14.1-143.89 | |
| pulmonary fibrosis | 87 | 16.55 | 13.37-20.48 | 16.41 | 13.28-20.26 | |
| wrong technique in device usage process | 358 | 14.82 | 13.32-16.49 | 14.25 | 12.86-15.79 | |
| lung lobectomy | 4 | 35.80 | 13.16-97.38 | 35.78 | 13.16-97.3 | |
| cholelithotomy | 3 | 41.37 | 12.98-131.86 | 41.35 | 12.98-131.77 | |
| dependence on oxygen therapy | 5 | 31.16 | 12.76-76.09 | 31.14 | 12.76-76.02 | |
| flatulence | 251 | 14.09 | 12.42-15.99 | 13.74 | 12.15-15.54 | |
| oxygen consumption decreased | 7 | 25.39 | 11.97-53.86 | 25.37 | 11.96-53.8 | |
| oxygen saturation decreased | 209 | 13.68 | 11.92-15.71 | 13.4 | 11.71-15.34 | |
| post procedural pneumonia | 3 | 37.66 | 11.84-119.76 | 37.65 | 11.84-119.68 | |
| total lung capacity abnormal | 3 | 37.66 | 11.84-119.76 | 37.65 | 11.84-119.68 | |
| small cell lung cancer | 12 | 20.08 | 11.33-35.62 | 20.06 | 11.32-35.55 | |
| pulmonary resection | 3 | 35.54 | 11.19-112.85 | 35.53 | 11.19-112.78 | |
| pneumothorax spontaneous | 7 | 23.10 | 10.9-48.96 | 23.08 | 10.9-48.9 | |
| diarrhoea haemorrhagic | 41 | 14.48 | 10.63-19.73 | 14.42 | 10.6-19.63 | |
| clubbing | 4 | 28.76 | 10.61-77.93 | 28.75 | 10.61-77.86 | |
| gastrointestinal sounds abnormal | 25 | 15.67 | 10.54-23.28 | 15.63 | 10.53-23.2 | |
| hypersensitivity pneumonitis | 8 | 21.10 | 10.46-42.58 | 21.09 | 10.46-42.52 | |
| decreased appetite | 823 | 11.10 | 10.33-11.93 | 10.23 | 9.58-10.92 | |
| pulmonary function test abnormal | 12 | 18.30 | 10.33-32.44 | 18.28 | 10.32-32.37 | |
| pulmonary pain | 18 | 16.43 | 10.3-26.2 | 16.4 | 10.29-26.13 | |
| haemorrhagic diathesis | 21 | 15.65 | 10.16-24.1 | 15.61 | 10.14-24.03 | |
| increased viscosity of bronchial secretion | 4 | 27.36 | 10.1-74.07 | 27.34 | 10.1-74 | |
| intestinal haemorrhage | 26 | 14.75 | 10-21.75 | 14.71 | 9.99-21.67 | |
| pneumonia bacterial | 36 | 12.92 | 9.29-17.96 | 12.87 | 9.27-17.88 | |
| haemorrhoidal haemorrhage | 23 | 13.85 | 9.17-20.92 | 13.81 | 9.15-20.85 | |
| bronchial carcinoma | 5 | 22.14 | 9.11-53.82 | 22.13 | 9.11-53.76 | |
| haemoptysis | 98 | 10.59 | 8.66-12.93 | 10.49 | 8.6-12.79 | |
| aortic valve calcification | 3 | 26.84 | 8.5-84.75 | 26.84 | 8.5-84.7 | |
| gastric disorder | 186 | 9.74 | 8.41-11.27 | 9.57 | 8.29-11.04 | |
| squamous cell carcinoma of lung | 6 | 18.84 | 8.39-42.31 | 18.83 | 8.38-42.27 | |
| weight decreased | 766 | 8.98 | 8.34-9.67 | 8.34 | 7.79-8.92 | |
| faeces discoloured | 73 | 10.01 | 7.94-12.62 | 9.94 | 7.9-12.51 | |
| large intestinal haemorrhage | 11 | 14.38 | 7.92-26.1 | 14.36 | 7.92-26.06 | |
| subcutaneous emphysema | 6 | 17.53 | 7.81-39.35 | 17.52 | 7.81-39.31 | |
| pulmonary hypertension | 74 | 9.78 | 7.77-12.31 | 9.71 | 7.73-12.2 | |
| secretion discharge | 43 | 10.43 | 7.72-14.1 | 10.39 | 7.69-14.02 | |
| haematochezia | 161 | 8.99 | 7.69-10.52 | 8.86 | 7.59-10.33 | |
| taste disorder | 67 | 9.73 | 7.64-12.39 | 9.67 | 7.61-12.29 | |
| hepatic enzyme increased | 182 | 8.34 | 7.2-9.67 | 8.2 | 7.1-9.48 | |
| cough | 702 | 7.57 | 7.01-8.18 | 7.08 | 6.59-7.61 | |
| rectal haemorrhage | 118 | 8.36 | 6.97-10.04 | 8.27 | 6.91-9.9 | |
| urinary bladder haemorrhage | 10 | 12.89 | 6.9-24.08 | 12.88 | 6.9-24.04 | |
| epistaxis | 222 | 7.86 | 6.88-8.99 | 7.7 | 6.76-8.78 | |
| interstitial lung disease | 130 | 7.96 | 6.69-9.48 | 7.87 | 6.63-9.34 | |
| sputum discoloured | 29 | 9.63 | 6.68-13.9 | 9.61 | 6.67-13.85 | |
| aortic rupture | 3 | 20.68 | 6.58-65.03 | 20.68 | 6.58-64.99 | |
| abdominal pain upper | 507 | 7.14 | 6.52-7.81 | 6.81 | 6.25-7.41 | |
| constipation | 509 | 7.10 | 6.49-7.76 | 6.77 | 6.22-7.37 | |
| rales | 17 | 10.42 | 6.46-16.82 | 10.4 | 6.45-16.78 | |
| respiratory failure | 172 | 7.50 | 6.45-8.73 | 7.38 | 6.36-8.57 | |
| sputum retention | 4 | 16.99 | 6.31-45.74 | 16.99 | 6.31-45.7 | |
| liver function test increased | 79 | 7.85 | 6.29-9.81 | 7.79 | 6.25-9.72 | |
| oxygen therapy | 8 | 12.58 | 6.26-25.3 | 12.57 | 6.26-25.27 | |
| gamma-glutamyltransferase abnormal | 3 | 18.69 | 5.95-58.69 | 18.69 | 5.95-58.66 | |
| increased tendency to bruise | 26 | 8.66 | 5.88-12.74 | 8.63 | 5.87-12.7 | |
| chromaturia | 58 | 7.56 | 5.83-9.8 | 7.52 | 5.81-9.73 | |
| gastric haemorrhage | 41 | 7.90 | 5.8-10.75 | 7.87 | 5.79-10.7 | |
| solar lentigo | 5 | 14.02 | 5.79-33.94 | 14.01 | 5.79-33.91 | |
| respiration abnormal | 24 | 8.52 | 5.7-12.75 | 8.5 | 5.69-12.71 | |
| pulmonary haemorrhage | 22 | 8.65 | 5.68-13.17 | 8.63 | 5.67-13.13 | |
| abdominal discomfort | 418 | 6.23 | 5.65-6.87 | 6 | 5.46-6.59 | |
| liver disorder | 107 | 6.76 | 5.58-8.18 | 6.69 | 5.54-8.08 | |
| walking distance test abnormal | 3 | 17.17 | 5.47-53.85 | 17.16 | 5.47-53.81 | |
| haemorrhoids | 42 | 7.35 | 5.42-9.96 | 7.32 | 5.4-9.91 | |
| dyspnoea | 1045 | 5.74 | 5.38-6.12 | 5.22 | 4.93-5.53 | |
| catheterisation cardiac | 16 | 8.69 | 5.31-14.22 | 8.67 | 5.3-14.19 | |
| scleroderma | 14 | 8.96 | 5.29-15.18 | 8.95 | 5.29-15.15 | |
| respiratory tract congestion | 33 | 7.45 | 5.29-10.5 | 7.43 | 5.28-10.46 | |
| dehydration | 226 | 5.95 | 5.22-6.8 | 5.84 | 5.13-6.64 | |
| multiple allergies | 23 | 7.86 | 5.21-11.86 | 7.85 | 5.21-11.82 | |
| discoloured vomit | 5 | 12.59 | 5.21-30.46 | 12.59 | 5.21-30.43 | |
| non-small cell lung cancer | 14 | 8.79 | 5.19-14.89 | 8.78 | 5.19-14.86 | |
| ageusia | 55 | 6.70 | 5.13-8.74 | 6.66 | 5.11-8.68 | |
| gastrointestinal disorder | 182 | 5.93 | 5.12-6.87 | 5.84 | 5.05-6.75 | |
| diverticulitis | 61 | 6.49 | 5.04-8.35 | 6.45 | 5.02-8.3 | |
| blood electrolytes decreased | 5 | 11.72 | 4.85-28.33 | 11.71 | 4.85-28.3 | |
| cardiac ablation | 7 | 10.19 | 4.83-21.48 | 10.18 | 4.83-21.45 | |
| frequent bowel movements | 49 | 6.37 | 4.81-8.45 | 6.34 | 4.79-8.4 | |
| ulcer haemorrhage | 19 | 7.53 | 4.79-11.84 | 7.52 | 4.79-11.81 | |
| paranasal sinus hypersecretion | 10 | 8.83 | 4.73-16.47 | 8.82 | 4.73-16.45 | |
| carbon dioxide increased | 5 | 11.37 | 4.7-27.48 | 11.36 | 4.7-27.46 | |
| gallbladder operation | 10 | 8.69 | 4.66-16.2 | 8.68 | 4.66-16.17 | |
| wrong dose | 11 | 8.39 | 4.63-15.19 | 8.38 | 4.63-15.17 | |
| hypoxia | 64 | 5.92 | 4.62-7.57 | 5.88 | 4.61-7.52 | |
| internal haemorrhage | 48 | 6.11 | 4.6-8.13 | 6.09 | 4.59-8.08 | |
| hepatic enzyme abnormal | 15 | 7.64 | 4.59-12.71 | 7.63 | 4.59-12.68 | |
| diet refusal | 3 | 14.26 | 4.55-44.63 | 14.25 | 4.55-44.61 | |
| pneumonia respiratory syncytial viral | 3 | 14.26 | 4.55-44.63 | 14.25 | 4.55-44.61 | |
| stent placement | 19 | 7.08 | 4.5-11.12 | 7.06 | 4.5-11.09 | |
| nausea | 1243 | 4.76 | 4.48-5.05 | 4.27 | 4.05-4.49 | |
| mucosal discolouration | 3 | 14.02 | 4.48-43.88 | 14.01 | 4.48-43.86 | |
| vomiting | 733 | 4.80 | 4.45-5.18 | 4.51 | 4.21-4.83 | |
| lung carcinoma cell type unspecified recurrent | 3 | 13.94 | 4.45-43.64 | 13.94 | 4.45-43.61 | |
| lung operation | 4 | 11.93 | 4.45-32.02 | 11.93 | 4.45-31.99 | |
| acute respiratory failure | 38 | 6.03 | 4.38-8.3 | 6.01 | 4.37-8.26 | |
| retching | 42 | 5.91 | 4.36-8.02 | 5.89 | 4.35-7.98 | |
| gastric perforation | 9 | 8.40 | 4.35-16.2 | 8.39 | 4.35-16.17 | |
| musculoskeletal chest pain | 29 | 6.26 | 4.34-9.02 | 6.24 | 4.33-8.99 | |
| lung disorder | 91 | 5.24 | 4.26-6.45 | 5.2 | 4.24-6.38 | |
| motion sickness | 6 | 9.52 | 4.26-21.3 | 9.52 | 4.26-21.28 | |
| pulmonary thrombosis | 26 | 6.23 | 4.23-9.16 | 6.21 | 4.23-9.13 | |
| large intestine infection | 4 | 11.22 | 4.18-30.09 | 11.21 | 4.18-30.06 | |
| blood urine present | 37 | 5.58 | 4.04-7.71 | 5.56 | 4.03-7.68 | |
| appetite disorder | 15 | 6.66 | 4.01-11.08 | 6.65 | 4-11.05 | |
| respiratory tract haemorrhage | 3 | 12.49 | 3.99-39.06 | 12.49 | 3.99-39.04 | |
| biliary obstruction | 4 | 10.68 | 3.98-28.64 | 10.68 | 3.98-28.62 | |
| intestinal perforation | 22 | 5.97 | 3.93-9.09 | 5.96 | 3.92-9.06 | |
| dysentery | 7 | 8.25 | 3.92-17.37 | 8.24 | 3.92-17.35 | |
| hypophagia | 40 | 5.17 | 3.79-7.06 | 5.15 | 3.78-7.03 | |
| diverticulum intestinal haemorrhagic | 8 | 7.58 | 3.78-15.21 | 7.58 | 3.78-15.19 | |
| gastrointestinal motility disorder | 12 | 6.63 | 3.76-11.71 | 6.63 | 3.76-11.69 | |
| aortic arteriosclerosis | 5 | 9.07 | 3.75-21.89 | 9.06 | 3.75-21.87 | |
| bowel movement irregularity | 17 | 6.04 | 3.75-9.74 | 6.03 | 3.74-9.71 | |
| transient ischaemic attack | 47 | 4.99 | 3.74-6.65 | 4.97 | 3.73-6.62 | |
| liver injury | 43 | 5.00 | 3.7-6.75 | 4.98 | 3.69-6.72 | |
| asthenia | 485 | 4.03 | 3.67-4.41 | 3.87 | 3.55-4.22 | |
| lung infection | 32 | 5.19 | 3.66-7.35 | 5.18 | 3.66-7.32 | |
| aspartate aminotransferase abnormal | 4 | 9.70 | 3.62-25.99 | 9.69 | 3.62-25.97 | |
| cor pulmonale | 3 | 11.26 | 3.61-35.2 | 11.26 | 3.61-35.18 | |
| gastrointestinal perforation | 11 | 6.43 | 3.55-11.64 | 6.42 | 3.55-11.62 | |
| illness | 141 | 4.11 | 3.48-4.86 | 4.06 | 3.45-4.79 | |
| cardiac ventricular thrombosis | 4 | 9.32 | 3.48-24.97 | 9.32 | 3.48-24.95 | |
| respiratory disorder | 46 | 4.64 | 3.47-6.2 | 4.62 | 3.46-6.17 | |
| pneumonia | 396 | 3.81 | 3.45-4.22 | 3.7 | 3.36-4.07 | |
| fibrosis | 7 | 7.24 | 3.44-15.25 | 7.24 | 3.44-15.23 | |
| dyspepsia | 136 | 4.05 | 3.42-4.8 | 4 | 3.39-4.73 | |
| hepatotoxicity | 44 | 4.59 | 3.41-6.18 | 4.58 | 3.41-6.15 | |
| gastrooesophageal reflux disease | 104 | 4.09 | 3.37-4.97 | 4.06 | 3.35-4.92 | |
| oesophageal rupture | 3 | 10.47 | 3.35-32.7 | 10.47 | 3.35-32.68 | |
| nephrotic syndrome | 14 | 5.65 | 3.34-9.56 | 5.64 | 3.34-9.54 | |
| pulmonary alveolar haemorrhage | 12 | 5.88 | 3.33-10.37 | 5.87 | 3.33-10.35 | |
| abdominal distension | 133 | 3.92 | 3.3-4.65 | 3.88 | 3.27-4.59 | |
| pulmonary sepsis | 4 | 8.69 | 3.25-23.29 | 8.69 | 3.25-23.27 | |
| pulmonary embolism | 117 | 3.88 | 3.23-4.66 | 3.85 | 3.21-4.61 | |
| hepatic function abnormal | 54 | 4.22 | 3.23-5.52 | 4.2 | 3.22-5.49 | |
| adenocarcinoma | 6 | 7.21 | 3.23-16.11 | 7.21 | 3.23-16.09 | |
| bile duct cancer | 4 | 8.63 | 3.22-23.11 | 8.62 | 3.22-23.09 | |
| abdominal pain | 261 | 3.63 | 3.21-4.11 | 3.56 | 3.16-4.01 | |
| carotid artery disease | 3 | 10.01 | 3.21-31.26 | 10.01 | 3.21-31.24 | |
| regurgitation | 7 | 6.73 | 3.2-14.17 | 6.73 | 3.2-14.15 | |
| pancreatic failure | 4 | 8.56 | 3.2-22.93 | 8.56 | 3.2-22.91 | |
| gamma-glutamyltransferase increased | 26 | 4.69 | 3.19-6.9 | 4.68 | 3.19-6.88 | |
| eating disorder | 36 | 4.42 | 3.19-6.14 | 4.41 | 3.18-6.12 | |
| chest pain | 204 | 3.64 | 3.16-4.18 | 3.58 | 3.12-4.1 | |
| hepatic cirrhosis | 29 | 4.51 | 3.13-6.5 | 4.5 | 3.13-6.48 | |
| anal haemorrhage | 6 | 6.97 | 3.12-15.57 | 6.97 | 3.12-15.56 | |
| colitis ischaemic | 12 | 5.51 | 3.12-9.72 | 5.5 | 3.12-9.7 | |
| cardiac pacemaker insertion | 11 | 5.56 | 3.07-10.06 | 5.55 | 3.07-10.04 | |
| dental operation | 6 | 6.77 | 3.03-15.11 | 6.76 | 3.03-15.1 | |
| transaminases increased | 36 | 4.20 | 3.03-5.84 | 4.19 | 3.02-5.81 | |
| testicular disorder | 3 | 9.45 | 3.03-29.49 | 9.45 | 3.03-29.48 | |
| increased viscosity of upper respiratory secretion | 3 | 9.42 | 3.02-29.38 | 9.41 | 3.02-29.36 | |
| lung adenocarcinoma | 7 | 6.31 | 3-13.27 | 6.3 | 3-13.25 | |
| anal incontinence | 17 | 4.80 | 2.98-7.73 | 4.79 | 2.97-7.71 | |
| intestinal ischaemia | 10 | 5.51 | 2.96-10.27 | 5.51 | 2.96-10.25 | |
| blood potassium decreased | 35 | 4.11 | 2.95-5.74 | 4.1 | 2.95-5.72 | |
| coronary artery occlusion | 14 | 4.99 | 2.95-8.43 | 4.98 | 2.95-8.42 | |
| right ventricular failure | 13 | 5.07 | 2.94-8.74 | 5.06 | 2.93-8.73 | |
| respiratory tract infection | 35 | 4.08 | 2.92-5.68 | 4.06 | 2.92-5.66 | |
| gastric ulcer haemorrhage | 10 | 5.38 | 2.89-10.02 | 5.38 | 2.89-10.01 | |
| seasonal allergy | 22 | 4.39 | 2.89-6.68 | 4.38 | 2.88-6.66 | |
| gastrointestinal infection | 13 | 4.97 | 2.88-8.57 | 4.96 | 2.88-8.56 | |
| eructation | 24 | 4.30 | 2.88-6.42 | 4.29 | 2.87-6.41 | |
| malignant neoplasm progression | 145 | 3.35 | 2.84-3.95 | 3.32 | 2.82-3.9 | |
| coronary arterial stent insertion | 12 | 4.96 | 2.81-8.75 | 4.96 | 2.81-8.74 | |
| gastrointestinal pain | 17 | 4.52 | 2.8-7.28 | 4.51 | 2.8-7.26 | |
| sjogren's syndrome | 9 | 5.36 | 2.78-10.33 | 5.36 | 2.78-10.32 | |
| painful respiration | 5 | 6.71 | 2.78-16.18 | 6.71 | 2.78-16.16 | |
| transplant | 7 | 5.77 | 2.74-12.15 | 5.77 | 2.74-12.13 | |
| colitis | 44 | 3.69 | 2.74-4.97 | 3.68 | 2.74-4.95 | |
| cholecystectomy | 13 | 4.71 | 2.73-8.12 | 4.7 | 2.73-8.11 | |
| hiatus hernia | 16 | 4.46 | 2.73-7.29 | 4.45 | 2.73-7.28 | |
| covid-19 pneumonia | 21 | 4.13 | 2.69-6.35 | 4.13 | 2.69-6.33 | |
| cerebral haemorrhage | 52 | 3.52 | 2.68-4.62 | 3.5 | 2.67-4.6 | |
| performance status decreased | 8 | 5.33 | 2.66-10.69 | 5.33 | 2.66-10.68 | |
| sudden death | 14 | 4.50 | 2.66-7.61 | 4.49 | 2.66-7.59 | |
| hepatitis toxic | 5 | 6.41 | 2.66-15.46 | 6.41 | 2.66-15.45 | |
| traumatic lung injury | 5 | 6.35 | 2.64-15.32 | 6.35 | 2.64-15.31 | |
| near death experience | 8 | 5.27 | 2.63-10.57 | 5.27 | 2.63-10.55 | |
| coronavirus infection | 15 | 4.36 | 2.62-7.24 | 4.35 | 2.62-7.23 | |
| cholelithiasis | 29 | 3.78 | 2.62-5.44 | 3.77 | 2.62-5.42 | |
| aphonia | 20 | 4.04 | 2.6-6.28 | 4.04 | 2.6-6.26 | |
| blood blister | 6 | 5.80 | 2.6-12.94 | 5.79 | 2.6-12.93 | |
| blood sodium decreased | 21 | 3.87 | 2.52-5.94 | 3.86 | 2.52-5.93 | |
| feeding disorder | 28 | 3.63 | 2.51-5.27 | 3.63 | 2.5-5.25 | |
| liver function test abnormal | 30 | 3.58 | 2.5-5.12 | 3.57 | 2.49-5.1 | |
| gallbladder disorder | 17 | 4.02 | 2.5-6.48 | 4.02 | 2.49-6.46 | |
| cardiac operation | 14 | 4.22 | 2.49-7.14 | 4.21 | 2.49-7.12 | |
| haematemesis | 31 | 3.55 | 2.49-5.05 | 3.54 | 2.49-5.04 | |
| haemorrhagic disorder | 3 | 7.76 | 2.49-24.2 | 7.76 | 2.49-24.19 | |
| sinus operation | 5 | 5.99 | 2.49-14.44 | 5.99 | 2.49-14.43 | |
| hypersomnia | 35 | 3.42 | 2.45-4.76 | 3.41 | 2.45-4.75 | |
| upper-airway cough syndrome | 13 | 4.22 | 2.45-7.29 | 4.22 | 2.45-7.28 | |
| pulmonary mass | 19 | 3.83 | 2.44-6.01 | 3.82 | 2.44-6 | |
| lung neoplasm | 5 | 5.87 | 2.43-14.14 | 5.86 | 2.43-14.13 | |
| intestinal mass | 3 | 7.58 | 2.43-23.62 | 7.58 | 2.43-23.6 | |
| enzyme level increased | 3 | 7.51 | 2.41-23.41 | 7.51 | 2.41-23.39 | |
| facial bones fracture | 7 | 5.05 | 2.4-10.61 | 5.04 | 2.4-10.6 | |
| covid-19 | 200 | 2.74 | 2.38-3.15 | 2.7 | 2.36-3.1 | |
| dysphonia | 61 | 3.05 | 2.37-3.92 | 3.03 | 2.36-3.9 | |
| contusion | 96 | 2.87 | 2.35-3.51 | 2.85 | 2.34-3.48 | |
| gastrointestinal haemorrhage | 115 | 2.82 | 2.35-3.39 | 2.8 | 2.33-3.36 | |
| acute coronary syndrome | 12 | 4.13 | 2.34-7.29 | 4.13 | 2.34-7.28 | |
| pneumonia viral | 5 | 5.64 | 2.34-13.59 | 5.64 | 2.34-13.58 | |
| death | 993 | 2.48 | 2.32-2.65 | 2.32 | 2.19-2.46 | |
| arterial occlusive disease | 9 | 4.47 | 2.32-8.6 | 4.46 | 2.32-8.59 | |
| lumbar spinal stenosis | 4 | 6.16 | 2.3-16.48 | 6.16 | 2.3-16.47 | |
| enterocolitis | 9 | 4.42 | 2.3-8.52 | 4.42 | 2.3-8.51 | |
| fatigue | 708 | 2.47 | 2.28-2.66 | 2.36 | 2.19-2.53 | |
| disease complication | 8 | 4.57 | 2.28-9.16 | 4.57 | 2.28-9.15 | |
| pneumatosis intestinalis | 6 | 5.05 | 2.26-11.28 | 5.05 | 2.26-11.27 | |
| epigastric discomfort | 7 | 4.75 | 2.26-9.98 | 4.74 | 2.26-9.97 | |
| dry mouth | 78 | 2.81 | 2.25-3.51 | 2.79 | 2.24-3.49 | |
| dysgeusia | 69 | 2.83 | 2.23-3.59 | 2.82 | 2.23-3.57 | |
| post procedural infection | 10 | 4.15 | 2.23-7.72 | 4.14 | 2.23-7.71 | |
| emphysema | 12 | 3.92 | 2.22-6.92 | 3.92 | 2.22-6.91 | |
| colectomy | 7 | 4.66 | 2.21-9.79 | 4.65 | 2.21-9.78 | |
| diverticular perforation | 4 | 5.90 | 2.21-15.79 | 5.9 | 2.21-15.77 | |
| bronchitis | 66 | 2.80 | 2.2-3.57 | 2.79 | 2.19-3.55 | |
| calculus bladder | 3 | 6.86 | 2.2-21.36 | 6.85 | 2.2-21.35 | |
| drug intolerance | 118 | 2.60 | 2.17-3.12 | 2.58 | 2.16-3.09 | |
| heart valve incompetence | 5 | 5.17 | 2.15-12.47 | 5.17 | 2.15-12.46 | |
| general physical health deterioration | 94 | 2.61 | 2.13-3.2 | 2.6 | 2.12-3.17 | |
| atrioventricular block | 11 | 3.85 | 2.13-6.97 | 3.85 | 2.13-6.96 | |
| urine odour abnormal | 9 | 4.08 | 2.12-7.85 | 4.08 | 2.12-7.84 | |
| aspiration | 12 | 3.73 | 2.12-6.58 | 3.73 | 2.12-6.57 | |
| cholecystitis | 12 | 3.73 | 2.12-6.58 | 3.73 | 2.11-6.57 | |
| laziness | 4 | 5.63 | 2.1-15.04 | 5.62 | 2.1-15.03 | |
| eye operation | 7 | 4.42 | 2.1-9.3 | 4.42 | 2.1-9.28 | |
| faeces pale | 4 | 5.58 | 2.09-14.92 | 5.58 | 2.09-14.91 | |
| blood magnesium decreased | 10 | 3.86 | 2.08-7.19 | 3.86 | 2.07-7.18 | |
| rhinorrhoea | 64 | 2.65 | 2.07-3.39 | 2.64 | 2.07-3.37 | |
| respiratory rate decreased | 5 | 4.95 | 2.05-11.92 | 4.95 | 2.05-11.91 | |
| hepatitis | 25 | 3.03 | 2.05-4.49 | 3.03 | 2.04-4.48 | |
| oesophageal haemorrhage | 3 | 6.32 | 2.03-19.7 | 6.32 | 2.03-19.68 | |
| sinus headache | 7 | 4.25 | 2.02-8.93 | 4.24 | 2.02-8.92 | |
| hypertension | 164 | 2.35 | 2.02-2.75 | 2.33 | 2-2.71 | |
| bronchial disorder | 3 | 6.25 | 2.01-19.45 | 6.24 | 2.01-19.44 | |
| blood pressure increased | 124 | 2.39 | 2-2.85 | 2.37 | 1.99-2.82 | |
| proctalgia | 7 | 4.20 | 2-8.83 | 4.2 | 2-8.82 | |
| metastases to central nervous system | 16 | 3.26 | 1.99-5.32 | 3.25 | 1.99-5.31 | |
| haemoglobin abnormal | 9 | 3.83 | 1.99-7.38 | 3.83 | 1.99-7.37 | |
| blood alkaline phosphatase increased | 19 | 3.11 | 1.98-4.88 | 3.11 | 1.98-4.87 | |
| hernia | 20 | 3.07 | 1.98-4.76 | 3.06 | 1.98-4.75 | |
| chest discomfort | 80 | 2.45 | 1.97-3.06 | 2.44 | 1.96-3.04 | |
| cystitis | 29 | 2.82 | 1.96-4.07 | 2.82 | 1.96-4.05 | |
| urine abnormality | 6 | 4.37 | 1.96-9.74 | 4.36 | 1.96-9.73 | |
| inflammatory bowel disease | 7 | 4.07 | 1.94-8.56 | 4.07 | 1.94-8.55 | |
| neutropenic sepsis | 9 | 3.72 | 1.93-7.17 | 3.72 | 1.93-7.16 | |
| pneumonia pseudomonal | 3 | 6.01 | 1.93-18.71 | 6.01 | 1.93-18.69 | |
| dry throat | 12 | 3.35 | 1.9-5.91 | 3.35 | 1.9-5.91 | |
| aneurysm | 7 | 3.97 | 1.89-8.35 | 3.97 | 1.89-8.34 | |
| haematuria | 35 | 2.62 | 1.88-3.65 | 2.61 | 1.88-3.64 | |
| colon cancer | 19 | 2.92 | 1.86-4.59 | 2.92 | 1.86-4.58 | |
| cerebrovascular accident | 136 | 2.20 | 1.85-2.6 | 2.18 | 1.84-2.58 | |
| sinus disorder | 21 | 2.84 | 1.85-4.36 | 2.84 | 1.85-4.35 | |
| glomerulonephritis rapidly progressive | 3 | 5.76 | 1.85-17.93 | 5.76 | 1.85-17.92 | |
| large intestine perforation | 8 | 3.70 | 1.85-7.42 | 3.7 | 1.85-7.41 | |
| blood pressure abnormal | 22 | 2.81 | 1.85-4.27 | 2.81 | 1.85-4.26 | |
| melaena | 24 | 2.76 | 1.85-4.12 | 2.75 | 1.85-4.11 | |
| blood bilirubin increased | 22 | 2.80 | 1.84-4.25 | 2.79 | 1.84-4.24 | |
| cardiac failure congestive | 65 | 2.34 | 1.83-2.99 | 2.33 | 1.83-2.97 | |
| nasopharyngitis | 140 | 2.16 | 1.83-2.55 | 2.14 | 1.82-2.53 | |
| abdominal pain lower | 23 | 2.75 | 1.83-4.15 | 2.75 | 1.83-4.14 | |
| muscle spasms | 143 | 2.16 | 1.83-2.54 | 2.14 | 1.82-2.52 | |
| abdominal rigidity | 4 | 4.88 | 1.83-13.05 | 4.88 | 1.83-13.04 | |
| renal pain | 12 | 3.21 | 1.82-5.66 | 3.21 | 1.82-5.65 | |
| cerebral infarction | 26 | 2.67 | 1.82-3.93 | 2.67 | 1.82-3.92 | |
| computerised tomogram abnormal | 3 | 5.66 | 1.82-17.61 | 5.66 | 1.82-17.6 | |
| aortic aneurysm | 12 | 3.15 | 1.79-5.56 | 3.15 | 1.79-5.55 | |
| cardiac failure | 67 | 2.28 | 1.79-2.89 | 2.27 | 1.78-2.88 | |
| abdominal tenderness | 6 | 3.96 | 1.77-8.83 | 3.96 | 1.77-8.82 | |
| faeces hard | 5 | 4.24 | 1.76-10.2 | 4.23 | 1.76-10.19 | |
| atrial fibrillation | 75 | 2.19 | 1.75-2.75 | 2.18 | 1.74-2.73 | |
| jaundice | 22 | 2.65 | 1.74-4.03 | 2.64 | 1.74-4.02 | |
| intestinal obstruction | 28 | 2.52 | 1.74-3.65 | 2.52 | 1.74-3.64 | |
| lung neoplasm malignant | 60 | 2.24 | 1.74-2.89 | 2.23 | 1.73-2.87 | |
| diverticulum | 8 | 3.48 | 1.74-6.97 | 3.48 | 1.74-6.96 | |
| anorectal discomfort | 5 | 4.17 | 1.73-10.05 | 4.17 | 1.73-10.04 | |
| duodenal ulcer | 8 | 3.45 | 1.72-6.91 | 3.45 | 1.72-6.91 | |
| pleural effusion | 45 | 2.31 | 1.72-3.1 | 2.3 | 1.72-3.08 | |
| oesophageal ulcer | 5 | 4.06 | 1.69-9.79 | 4.06 | 1.69-9.78 | |
| nephrolithiasis | 36 | 2.34 | 1.69-3.25 | 2.34 | 1.69-3.24 | |
| sepsis | 74 | 2.12 | 1.68-2.66 | 2.11 | 1.68-2.64 | |
| pneumonitis | 23 | 2.52 | 1.68-3.8 | 2.52 | 1.68-3.79 | |
| raynaud's phenomenon | 6 | 3.72 | 1.67-8.3 | 3.72 | 1.67-8.29 | |
| peripheral artery thrombosis | 3 | 5.18 | 1.66-16.12 | 5.18 | 1.67-16.11 | |
| post procedural haemorrhage | 11 | 2.99 | 1.65-5.4 | 2.98 | 1.65-5.39 | |
| exercise tolerance decreased | 7 | 3.43 | 1.63-7.2 | 3.43 | 1.63-7.2 | |
| oesophageal stenosis | 4 | 4.35 | 1.63-11.61 | 4.35 | 1.63-11.61 | |
| gastric ulcer | 17 | 2.57 | 1.6-4.14 | 2.57 | 1.6-4.14 | |
| feeling cold | 22 | 2.43 | 1.6-3.69 | 2.42 | 1.6-3.68 | |
| hepatic failure | 24 | 2.38 | 1.6-3.56 | 2.38 | 1.6-3.55 | |
| cancer pain | 4 | 4.24 | 1.59-11.32 | 4.24 | 1.59-11.31 | |
| hip fracture | 26 | 2.32 | 1.58-3.41 | 2.32 | 1.58-3.4 | |
| femoral neck fracture | 6 | 3.51 | 1.58-7.84 | 3.51 | 1.58-7.83 | |
| vomiting projectile | 4 | 4.20 | 1.57-11.21 | 4.19 | 1.57-11.2 | |
| increased appetite | 15 | 2.60 | 1.57-4.33 | 2.6 | 1.57-4.32 | |
| wheezing | 42 | 2.12 | 1.56-2.87 | 2.11 | 1.56-2.86 | |
| alanine aminotransferase abnormal | 3 | 4.82 | 1.55-15.01 | 4.82 | 1.55-15 | |
| surgery | 46 | 2.07 | 1.55-2.77 | 2.07 | 1.55-2.76 | |
| pericardial effusion | 19 | 2.43 | 1.55-3.82 | 2.43 | 1.55-3.81 | |
| acute myocardial infarction | 21 | 2.35 | 1.53-3.6 | 2.34 | 1.53-3.59 | |
| pulseless electrical activity | 6 | 3.39 | 1.52-7.55 | 3.39 | 1.52-7.55 | |
| respiratory symptom | 4 | 4.03 | 1.51-10.78 | 4.03 | 1.51-10.77 | |
| bronchitis chronic | 3 | 4.66 | 1.5-14.51 | 4.66 | 1.5-14.5 | |
| ischaemic stroke | 18 | 2.38 | 1.5-3.78 | 2.38 | 1.5-3.78 | |
| heart rate irregular | 17 | 2.41 | 1.5-3.88 | 2.41 | 1.5-3.88 | |
| vocal cord disorder | 3 | 4.66 | 1.5-14.48 | 4.65 | 1.5-14.47 | |
| pseudomembranous colitis | 3 | 4.61 | 1.48-14.35 | 4.61 | 1.48-14.34 | |
| retinal artery occlusion | 4 | 3.96 | 1.48-10.57 | 3.96 | 1.48-10.57 | |
| blood pressure fluctuation | 17 | 2.37 | 1.47-3.82 | 2.37 | 1.47-3.81 | |
| myocardial necrosis marker increased | 3 | 4.57 | 1.47-14.22 | 4.57 | 1.47-14.21 | |
| international normalised ratio abnormal | 5 | 3.54 | 1.47-8.52 | 3.54 | 1.47-8.51 | |
| malnutrition | 8 | 2.94 | 1.47-5.89 | 2.94 | 1.47-5.88 | |
| skin laceration | 9 | 2.83 | 1.47-5.44 | 2.82 | 1.47-5.43 | |
| electrolyte imbalance | 10 | 2.70 | 1.45-5.03 | 2.7 | 1.45-5.03 | |
| proctitis | 3 | 4.51 | 1.45-14.02 | 4.5 | 1.45-14.01 | |
| blood test abnormal | 14 | 2.44 | 1.45-4.13 | 2.44 | 1.45-4.12 | |
| pulmonary oedema | 30 | 2.07 | 1.44-2.96 | 2.06 | 1.44-2.95 | |
| blood iron decreased | 10 | 2.69 | 1.44-5 | 2.68 | 1.44-4.99 | |
| tension headache | 5 | 3.47 | 1.44-8.35 | 3.47 | 1.44-8.35 | |
| drug-induced liver injury | 29 | 2.07 | 1.44-2.98 | 2.07 | 1.44-2.97 | |
| cardiac valve disease | 6 | 3.20 | 1.44-7.14 | 3.2 | 1.44-7.13 | |
| gastroenteritis viral | 14 | 2.38 | 1.41-4.03 | 2.38 | 1.41-4.02 | |
| gout | 15 | 2.34 | 1.41-3.89 | 2.34 | 1.41-3.88 | |
| haemorrhage urinary tract | 4 | 3.74 | 1.4-9.98 | 3.74 | 1.4-9.98 | |
| decreased activity | 11 | 2.52 | 1.39-4.55 | 2.52 | 1.39-4.55 | |
| nasal dryness | 7 | 2.91 | 1.39-6.12 | 2.91 | 1.39-6.11 | |
| duodenal ulcer haemorrhage | 4 | 3.70 | 1.39-9.89 | 3.7 | 1.39-9.88 | |
| defaecation urgency | 6 | 3.08 | 1.38-6.87 | 3.08 | 1.38-6.87 | |
| portal vein thrombosis | 4 | 3.69 | 1.38-9.84 | 3.68 | 1.38-9.83 | |
| lip and/or oral cavity cancer | 3 | 4.29 | 1.38-13.35 | 4.29 | 1.38-13.34 | |
| rib fracture | 15 | 2.29 | 1.38-3.8 | 2.29 | 1.38-3.8 | |
| muscle strain | 8 | 2.76 | 1.38-5.52 | 2.76 | 1.38-5.52 | |
| lower gastrointestinal haemorrhage | 9 | 2.65 | 1.37-5.09 | 2.64 | 1.37-5.09 | |
| ulcer | 13 | 2.33 | 1.35-4.02 | 2.33 | 1.35-4.02 | |
| dysuria | 25 | 2.00 | 1.35-2.97 | 2 | 1.35-2.96 | |
| hepatic fibrosis | 4 | 3.59 | 1.34-9.58 | 3.59 | 1.34-9.57 | |
| large intestinal obstruction | 3 | 4.15 | 1.33-12.91 | 4.15 | 1.33-12.9 | |
| clostridium difficile infection | 16 | 2.18 | 1.33-3.56 | 2.18 | 1.33-3.55 | |
| volvulus | 3 | 4.14 | 1.33-12.88 | 4.14 | 1.33-12.88 | |
| haemothorax | 4 | 3.55 | 1.33-9.47 | 3.54 | 1.33-9.46 | |
| impaired healing | 17 | 2.14 | 1.33-3.44 | 2.13 | 1.33-3.43 | |
| white blood cell disorder | 4 | 3.52 | 1.32-9.4 | 3.52 | 1.32-9.39 | |
| coronary artery disease | 16 | 2.15 | 1.32-3.51 | 2.15 | 1.32-3.51 | |
| lung opacity | 3 | 4.03 | 1.3-12.53 | 4.03 | 1.3-12.53 | |
| renal function test abnormal | 5 | 3.11 | 1.29-7.48 | 3.11 | 1.29-7.48 | |
| oral candidiasis | 9 | 2.48 | 1.29-4.77 | 2.48 | 1.29-4.77 | |
| back injury | 8 | 2.56 | 1.28-5.13 | 2.56 | 1.28-5.12 | |
| small intestinal haemorrhage | 3 | 3.97 | 1.28-12.36 | 3.97 | 1.28-12.35 | |
| food poisoning | 5 | 3.07 | 1.28-7.39 | 3.07 | 1.28-7.39 | |
| acute lung injury | 3 | 3.97 | 1.28-12.34 | 3.97 | 1.28-12.33 | |
| procedural complication | 5 | 3.07 | 1.27-7.38 | 3.06 | 1.27-7.37 | |
| hunger | 9 | 2.44 | 1.27-4.69 | 2.44 | 1.27-4.69 | |
| cyanosis | 10 | 2.35 | 1.26-4.38 | 2.35 | 1.26-4.37 | |
| rhinovirus infection | 4 | 3.37 | 1.26-9 | 3.37 | 1.26-9 | |
| respiratory rate increased | 6 | 2.81 | 1.26-6.26 | 2.81 | 1.26-6.25 | |
| retinal tear | 3 | 3.91 | 1.26-12.16 | 3.91 | 1.26-12.16 | |
| tooth infection | 10 | 2.33 | 1.25-4.34 | 2.33 | 1.25-4.33 | |
| phlebitis | 4 | 3.33 | 1.25-8.89 | 3.33 | 1.25-8.89 | |
| head discomfort | 15 | 2.07 | 1.25-3.44 | 2.07 | 1.25-3.43 | |
| small intestinal obstruction | 8 | 2.49 | 1.25-4.99 | 2.49 | 1.25-4.99 | |
| abnormal faeces | 7 | 2.60 | 1.24-5.45 | 2.59 | 1.24-5.45 | |
| yellow skin | 5 | 2.96 | 1.23-7.13 | 2.96 | 1.23-7.13 | |
| sinus congestion | 9 | 2.37 | 1.23-4.55 | 2.37 | 1.23-4.55 | |
| foreign body in throat | 4 | 3.21 | 1.2-8.58 | 3.21 | 1.2-8.57 | |
| prostatomegaly | 4 | 3.16 | 1.18-8.43 | 3.16 | 1.18-8.42 | |
| metastases to meninges | 3 | 3.66 | 1.18-11.38 | 3.66 | 1.18-11.38 | |
| peripheral arterial occlusive disease | 4 | 3.11 | 1.16-8.3 | 3.11 | 1.16-8.3 | |
| colonoscopy | 3 | 3.58 | 1.15-11.14 | 3.58 | 1.15-11.13 | |
| eye contusion | 3 | 3.57 | 1.15-11.11 | 3.57 | 1.15-11.1 | |
| blood potassium increased | 11 | 2.07 | 1.15-3.75 | 2.07 | 1.15-3.74 | |
| peripheral ischaemia | 4 | 3.06 | 1.15-8.17 | 3.06 | 1.15-8.16 | |
| concussion | 6 | 2.55 | 1.14-5.67 | 2.54 | 1.14-5.67 | |
| intra-abdominal haemorrhage | 3 | 3.55 | 1.14-11.03 | 3.55 | 1.14-11.03 | |
| gastric infection | 4 | 3.00 | 1.12-8.01 | 3 | 1.12-8 | |
| wound dehiscence | 3 | 3.43 | 1.1-10.66 | 3.43 | 1.1-10.65 | |
| hip surgery | 5 | 2.63 | 1.09-6.33 | 2.63 | 1.09-6.32 | |
| tooth extraction | 8 | 2.17 | 1.09-4.35 | 2.17 | 1.09-4.35 | |
| cardiomegaly | 7 | 2.28 | 1.09-4.79 | 2.28 | 1.09-4.78 | |
| bile duct stone | 3 | 3.35 | 1.08-10.41 | 3.35 | 1.08-10.41 | |
| gingival bleeding | 9 | 2.07 | 1.07-3.98 | 2.07 | 1.07-3.97 | |
| retroperitoneal haemorrhage | 3 | 3.32 | 1.07-10.33 | 3.32 | 1.07-10.33 | |
| cerebral thrombosis | 3 | 3.32 | 1.07-10.32 | 3.32 | 1.07-10.31 | |
| cholangitis | 5 | 2.57 | 1.07-6.17 | 2.57 | 1.07-6.17 | |
| breast mass | 5 | 2.54 | 1.06-6.11 | 2.54 | 1.06-6.11 | |
| glomerulonephritis | 3 | 3.27 | 1.05-10.17 | 3.27 | 1.05-10.17 | |
| blood loss anaemia | 3 | 3.24 | 1.04-10.08 | 3.24 | 1.04-10.07 | |
| appendicectomy | 3 | 3.24 | 1.04-10.07 | 3.24 | 1.04-10.06 | |
| hepatic pain | 4 | 2.73 | 1.02-7.29 | 2.73 | 1.02-7.28 | |
| helicobacter infection | 5 | 2.45 | 1.02-5.9 | 2.45 | 1.02-5.89 | |
| immobile | 4 | 2.71 | 1.01-7.22 | 2.71 | 1.01-7.22 | |
| international normalised ratio fluctuation | 3 | 3.15 | 1.01-9.78 | 3.15 | 1.01-9.77 | |
| lung cancer metastatic | 3 | 3.13 | 1.01-9.74 | 3.13 | 1.01-9.73 | |
| heart rate abnormal | 5 | 2.41 | 1-5.8 | 2.41 | 1-5.79 | |

FAERS, Food and Drug Administration Adverse Event Reporting System; PTs, preferred terms; ROR, reporting odds ratio; 95% CI, 95% credibility interval.
